# Functional Connections in the Brain Transform Experience into Memory

**DOI:** 10.1371/journal.pbio.0040165

**Published:** 2006-04-18

**Authors:** Liza Gross

The ephemeral quality of memory is captured in a classic Steven Wright joke. “The other day I . . . no, wait,” he deadpans. “That wasn't me.” Most real-life bouts of episodic memory loss are more mundane—what did I have for dinner last night?—and perfectly normal. And considering the constant stream of stimuli most people encounter in a single day, it's a wonder anyone remembers anything.

To process new experiences, discrete regions in your brain transform a rich palette of perceptual information into an internal representation of the experience. Over time, different regions of the brain consolidate the neural traces of these episodes into more enduring memories. Behavioral and neuroimaging studies have implicated a network of brain structures—including the medial temporal lobes (MTL), prefrontal cortex (PFC), and sensory cortical areas—in episodic memory formation. How these memory centers collaborate, and particularly what role the PFC plays, remains a subject of debate.

Neuroimaging techniques are typically used to link brain activity with a particular task or function. In a new study, Christopher Summerfield, Jennifer Mangels, and colleagues take functional magnetic resonance imaging (fMRI) a step further to determine the functional connections across multiple brain regions during episodic memory processing. They show that functional links between the dorsolateral PFC and two regions of the sensory neocortex predict how well people remember associations between images of faces and houses.

They propose a model whereby the PFC exerts “top down” control over episodic memory processing by modulating activity in the sensory cortex to select which inputs go into a new memory trace. These inputs are selectively channeled through different stages of the processing hierarchy until they reach MTL structures, such as the hippocampus, for consolidation into long-term memory. Previous neuroimaging studies have shown that blood flow changes in areas of the PFC and sensory cortex vary as a function of what neuroscientists call “encoding success”—the likelihood of forming a new long-term memory. And activation of brain regions associated with learning new associations can often predict a participant's encoding success. But stronger support for this model, Summerfield et al. argue, would come from showing that functional connectivity between different brain regions in the processing hierarchy can also predict encoding success.

To look for evidence of functional connectivity, the researchers focused on two regions of the extrastriate visual cortex that selectively respond to faces—the fusiform face area (FFA)—and houses—the parahippocampal place area (PPA). In the first phase of each of 20 blocks (groups of stimuli), participants viewed seven consecutive pairs of faces and houses and were asked to memorize (or “intentionally encode”) associations between faces and houses. During the testing phase, participants viewed the original pairs, interspersed with seven new pairs, remixed from the original images. Participants indicated whether the pairs were old or new by pressing one of five buttons, indicating confidence in their choice. The authors predicted that FFA and PPA responses would track memory performance (encoding success), and that connectivity between the FFA/PPA regions and the PFC could predict performance.

A search for brain regions in which responses related to learning the associations differed from subsequent memory of the image pairs identified a region associated with early visual processing, the lateral occipital complex (LOC), as well as the FFA, PPA, and MTL. Later memory effects were also seen in the vascular response, with peak responses occurring progressively later in the processing hierarchy—a pattern that might reflect the ongoing processing of task-relevant perceptual codes that go on to the next stage of processing.

To determine functional connectivity, the researchers focused on state-related responses. State-related activity—rather than stimulus-evoked responses—they explain, may reflect ongoing brain activity that is not limited to a stimulus but helps regulate its integration into memory formation. By correlating encoding success with connectivity between the left dorsolateral PFC and the FFA/PPA, they showed that connectivity predicted later memory performance for each participant. They go on to show that connectivity between this PFC region was “reliably greater” than connectivity between the PFC and other regions in the processing hierarchy.

By showing that connectivity can predict behavioral performance, these results provide support for the top-down model in which the PFC regulates activity in lower cortical regions to cherry pick representations most relevant to the task at hand. This model may shed light on other modes of neurocognitive processing as well. The next big challenge will be to figure how individual neurons mediate these functional connections across multiple brain regions.

## 

**Figure:**
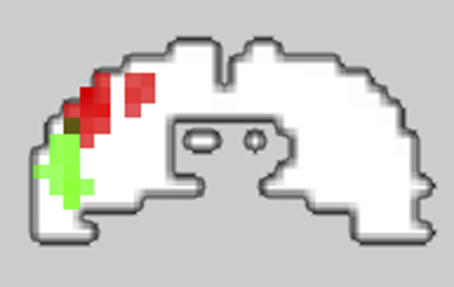
Regions of the dorsolateral prefrontal cortex found to exhibit significant connectivity with face-sensitive (red) and place-sensitive (green) visual regions during the formation of new associations between faces and places.

